# A short-term musical training affects implicit emotion regulation only in behaviour but not in brain activity

**DOI:** 10.1186/s12868-021-00636-1

**Published:** 2021-04-26

**Authors:** M. Berthold-Losleben, S. Papalini, U. Habel, K. Losleben, F. Schneider, K. Amunts, N. Kohn

**Affiliations:** 1 Department of Mental Health, Norwegian University of Science and Technology (NTNU), Trondheim, Norway; 2 Division of Mental Healthcare, St. Olavs University Hospital, Trondheim, Norway; 3 Laboratory for Biological Psychology, Brain and Cognition Unit, KU Leuven, Leuven, Belgium; 4 Department of Psychiatry, Psychotherapy and Psychosomatics, University Hospital RWTH, Aachen, Germany; 5 Centre for Women’s and Gender Studies, The Arctic University of Norway (UiT), Tromsø, Norway; 6 University Hospital Düsseldorf, Düsseldorf, Germany; 7 Institute of Neuroscience and Medicine, Jülich Research Centre, Jülich, Germany; 8 Cécile and Oskar Vogt Institute of Brain Research, Heinrich Heine University, University Hospital Düsseldorf, Düsseldorf, Germany; 9 Department for Cognitiv Neuroscience, Donders Institute for Brain, Cognition and Behaviour, Radboud University, Postbus 9101, 6500 HB Nijmegen, The Netherlands

**Keywords:** Music, Olfaction, FMRI, Emotion Regulation, Training, Affective Rivalry, Multisensory Integration, Listening, Implicit

## Abstract

**Background:**

In everyday life, negative emotions can be implicitly regulated by positive stimuli, without any conscious cognitive engagement; however, the effects of such implicit regulation on mood and related neuro-mechanisms, remain poorly investigated in literature. Yet, improving implicit emotional regulation could reduce psychological burden and therefore be clinically relevant for treating psychiatric disorders with strong affective symptomatology.

**Results:**

Music training reduced the negative emotional state elicited by negative odours. However, such change was not reflected at the brain level.

**Conclusions:**

In a context of affective rivalry a musical training enhances implicit regulatory processes. Our findings offer a first base for future studies on implicit emotion regulation in clinical populations.

## Background

The ability to effectively regulate emotions has direct impacts on the level of our well-being [1, 2]. Such ability constantly influences decision-making processes and has conspicuous consequences on our daily social interactions [3, 4]. Emotion regulation can be understood as an either implicit (automatic, without monitoring or insight) or explicit (monitored with conscious effort and awareness) regulatory process of affective internal states [5]. From a neurocognitive perspective, this regulation has historically been seen as the result of bottom-up or top-down brain activation processes, describing mainly cortical-subcortical interactions. Both carry salient information about our internal and/or external environment, and lead to a certain affective state and associated behavioural reactions [6–10]. However, newer views see regulation as a more complex interactive process on multiple levels [11, 12].

As a matter of fact, our affective state is often generated and updated by multisensory incoming stimuli which can be of conflicting nature [13]. In such circumstances, which we earlier referred to as a state of ‘affective rivalry’, we are usually confronted with difficulties in regulating and integrating affective states [13]. Difficulties in regulating emotions can indeed converge into dysregulated mood states that have the potential to contribute as risk factor to the development of affective disorders, such as pathological anxiety and clinical depression [14–16]. Hence, these difficulties might be particularly exacerbated in patients with a diagnosis of affective disorder. To eventually aid treatment of affective disorders we aim to train adaptive emotion regulation on incongruent emotional states, at first in healthy volunteers in the present study, as well as in clinical circumstances as subject of future research.

In a previous paper we presented a design that encompassed both, multisensory integration and affective rivalry, and investigated on a behavioural and functional brain level how implicit regulatory processes during incongruent emotional situations affected mood in healthy participants [13]. Although positive and negative music as well as pleasant and unpleasant odours are well established stimuli for independently inducing congruent emotions like happiness, sadness or disgust [17–21], to our knowledge no studies at that time had combined these modalities as rivals to investigate the resulting emotional state. To do so we recreated in our previous laboratory setting a state of “affective rivalry” with auditory (musical) and olfactory stimuli. We then confirmed that negative olfactory and positive auditory stimuli are independently capable of inducing congruent emotional states, and moreover demonstrated, that when pairing a negative olfactory with a positive auditory stimulus, the olfactory stimulus dominates the emotional response. The results we found were likely related to the unique “hard-wired” anatomic connections of the olfactory system and its evolutionary salience [22]. By contrast, the affective perception of music is assumed involves both automatic bottom-up and more cognitively influenced top-down regulation processes [19, 23, 24], but is being capable of inducing strong positive and negative emotions, and even emotionally neutral states [19, 25, 26]. Despite the strong potential to induce affectively valenced states, a strong interindividual variance in this effect has been observed [27, 28]. In this study we focus on the feeling state induced by emotions rather than a cognitive labelling, which has been separately described [29, 30].

The results from our previous study support the hypothesis that music can implicitly modulate the subjective experience of an existing negative affective state. The interaction of valenced music and valenced olfaction represents a regulatory process that is "uninstructed, effortless and proceeds without awareness", which is how implicit emotion regulation can be defined [5]. Commonly, implicit emotion regulation is investigated by fear inhibition and emotional conflict paradigms, which are associated to activity in ventral anterior cingulate and ventromedial prefrontal cortex [5, 6, 31]. To date, however, the presence of long-term effects of this modulation on mood remains to be investigated, at behavioural as well as the brain level [32]. These potential effects could be then used to contribute to a better stabilization of dysregulated emotional states. For instance, a musical intervention such as music-listening may be applied for the prevention as well as an adjunct to the classical treatments of affective disorders, in order to achieve better emotional regulation outcomes [33].

In the present study we investigated whether and to what extend music-listening was able to implicitly modulate emotion regulation during an affectively rival situation. To examine this hypothesis, we employ the same affectively rivalry design as in our previous study. Specifically, we investigated whether the regulatory effects of positive auditory stimuli on mood induction by unpleasant odour can be modulated by a three-week musical training, and to what extend such an experience can lead to functional changes at the brain level. We predicted a significant positive effect of the musical intervention on the individual mood levels. We anticipated that such change might reflect improved implicit emotion counter-regulation abilities, probably mediated by changes in brain activity in areas that have been previously associated with implicit emotion regulation or salience processing, such as (dorsal) anterior cingulate cortex, inferior frontal cortex, amygdala and ventromedial prefrontal cortex [6, 34]. According to our prediction that music-listening on a regular basis modulates emotion regulation and is capable of improving counter-regulation abilities, we refer to our intervention furthermore as musical training in the sense of passive music-listening and not active music-performing, while musical experience or simply music-listening also might be applicable terms.

## Results

### Behavioural results

The three ratings are reported successively: Disgust ratings to begin with, followed by odour ratings and finally the emotional subjective state ratings. Means and standard errors are reported in Table 1.

**Table 1:** Mean values and standard errors for the ratings of the **A** disgust level, **B** music valence, and **C** emotional state

	A^o^O^−^	A^o^O^o^	A^+^O^−^	A^+^O^o^
A Disgust level				
OS T0	4.01 (.12)	1.231 (.04)	4.036 (.12)	1.255 (.05)
OS T1	3.526 (.13)	1.122 (.04)	3.411 (.13)	1.119 (.04)
CG T0	3.989 (.19)	1.208 (0.6)	4.05 (.17)	1.213 (.05)
CG T1	3.677 (.19)	1.182 (.07)	3.562 (.19)	1.197 (.07)
TG T0	4.047 (.15)	1.255 (.08)	4.020 (.18)	1.296 (.08)
TG T1	3.375 (.18)	1.062 (.02)	3.260 (.18)	1.041 (.02)
B Music valence				
OS T0	2.898 (.11)	4.153 (.09)	3.805 (.093)	3.244 (.10)
OS T1	3.03 (.13)	4.247 (.076)	4.013 (.09)	3.246 (.11)
CG T0	3.072 (.15)	4.130 (.15)	3.81 (.15)	3.416 (.14)
CG T1	3.114 (.18)	4.187 (.11)	3.953 (.12)	3.359 (.15)
TG T0	2.724 (.15)	4.177 (.09)	3.797 (.11)	3.072 (.13)
TG T1	2.953 (.176)	4.307 (.10)	4.072 (.14)	3.133 (.16)
C Emotional state				
OS T0	2.679 (.08)	3.724 (.10)	2.843 (.10)	3.401 (.08)
OS T1	2.988 (.10)	3.903 (.07)	3.372 (.11)	3.427 (.08)
CG T0	2.750 (.12)	3.750 (.14)	2.890 (.13)	3.484 (.13)
CG T1	2.817 (.14)	3.822 (.09)	3.083 (.14)	3.375 (.09)
TG T0	2.609 (.12)	3.698 (.12)	2.786 (.14)	3.317 (.11)
TG T1	3.156 (.14)	3.984 (.09)	3.661 (.13)	3.479 (.13)

The columns show the results of the first (**T0**) and second (**T1**) measurement of the overall sample (**OS**; n=32), control group (**CG**; n=16) and training group (**TG**; n=16) for the four conditions: Neutral auditory and negative olfactory stimulation (**A**^**o**^**O**^**−**^), neutral auditory and neutral olfactory stimulation (**A**^**o**^**O**^**o**^), positive auditory and negative olfactory stimulation (**A**^**+**^**O**^**−**^), and positive auditory and neutral olfactory stimulation (**A**^**+**^**O**^**o**^)

#### Disgust ratings of olfactory stimuli

**Fig. 1:**
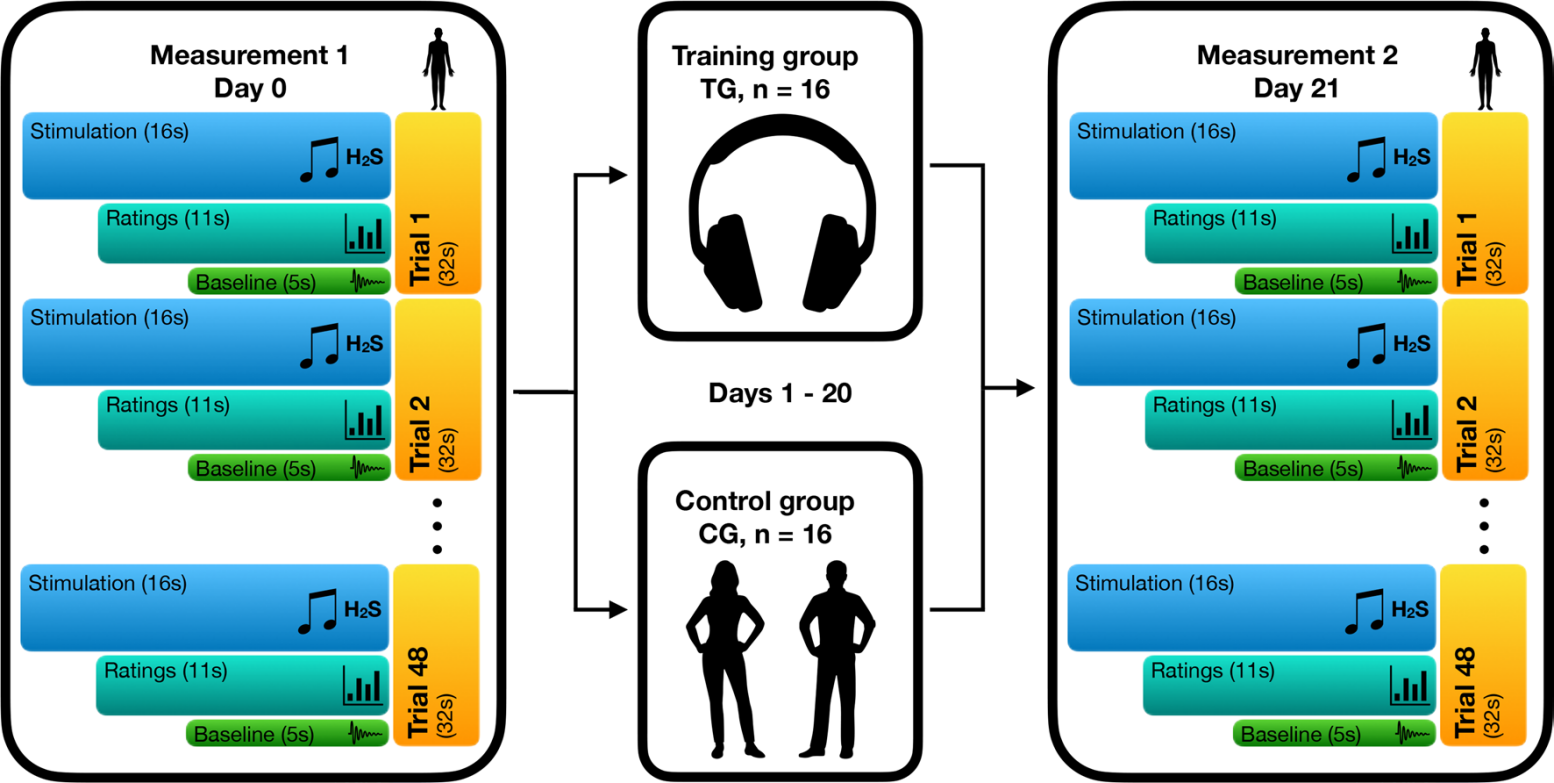
Results of the **a** disgust ratings of the olfactory stimuli, **b** the valence ratings of the auditory stimuli, and **C** the ratings of the emotional states. The ratings are shown for the first (**T0**) and the second (**T1**) measurement of the control group (**CG**) without an intervention and the training group (**TG**) which received a twenty-day musical training between the two measurements. The four conditions are **A**^**0**^**O**^**−**^ (neutral auditory combined with negative olfactory stimulation), **A**^**0**^**O**^**0**^ (neutral auditory combined with neutral olfactory stimulation), **A**^**+**^**O**^**−**^ (positive auditory combined with negative olfactory stimulation), and **A**^**+**^**O**^**0**^ (positive auditory combined with neutral olfactory stimulation)

The two groups did not show differences at the baseline in disgust rating scores (all p > 0.05). Negative odours stimuli were rated significantly more negative compared to the neutral odour stimuli (t = 22.375, p =  < 0.001). Before the training (T0) a significant effect of the negative olfactory stimuli (O) on disgust ratings across the sample was found (F_1,30_ = 556.13, p < 0.001, η_p_^2^ = 0.95) independently of the occurrence of neutral or positive auditory stimuli (A x O, p > 0.05). No significant effects of the positive auditory stimuli were found (A) (t = - 0.764, p = 0.45). At the end of the training (T1), a general effect of time (F_1,30_ = 32.55, p < 0.001, η_p_^2^ = 0.52), and a significant interaction between the effect of the negative olfactory stimuli over time (O x T) (F_1,30_ = 13.27, p = 0.001, η_p_^2^ = 0.31) was found. Post hoc results from paired t-tests indicated a reduction in disgust toward negative stimuli among the sample at the second measurement (t = 3.863, p = 0.001). The same interaction was not significant for auditory stimuli (A x T, p > 0.05). There was a significant difference in decrease of disgust rating over time between the two groups T x G (F_1,30_ = 4.71, p = 0.038, η_p_^2^ = 0.136), the training group in general showed a stronger decrease in disgust over all conditions. The more specific interaction effects were not significant (A x O x T, O x T x G or A x O x T x G) (all p > 0.05). The latter might be related to a floor effect in the conditions with no disgusting odour (O^o^). Figure 1a shows an overview of the disgust ratings.

#### Valence ratings of auditory stimuli

The two groups did not differ at baseline in auditory rating scores (all p > 0.05). In general, in both measurements, the effect of the auditory stimuli (A) on the valence ratings of the music was significant (F_1,30_ = 69.31, p < 0.001; η_p_^2^ = 0.69). Positive auditory stimuli were rated significantly more positive compared to neutral auditory stimuli (t = − 7.118, p < 0.001). A significant effect of the olfactory stimuli on the valence ratings of the music was also found (F_1,30_ = 67.61, p < 0.001; η_p_^2^ = 0.69). Specifically, positive auditory stimuli were rated as more pleasant during neutral compared with negative olfactory stimulation (t = − 5.974, p < 0.001).

**Fig. 2:**
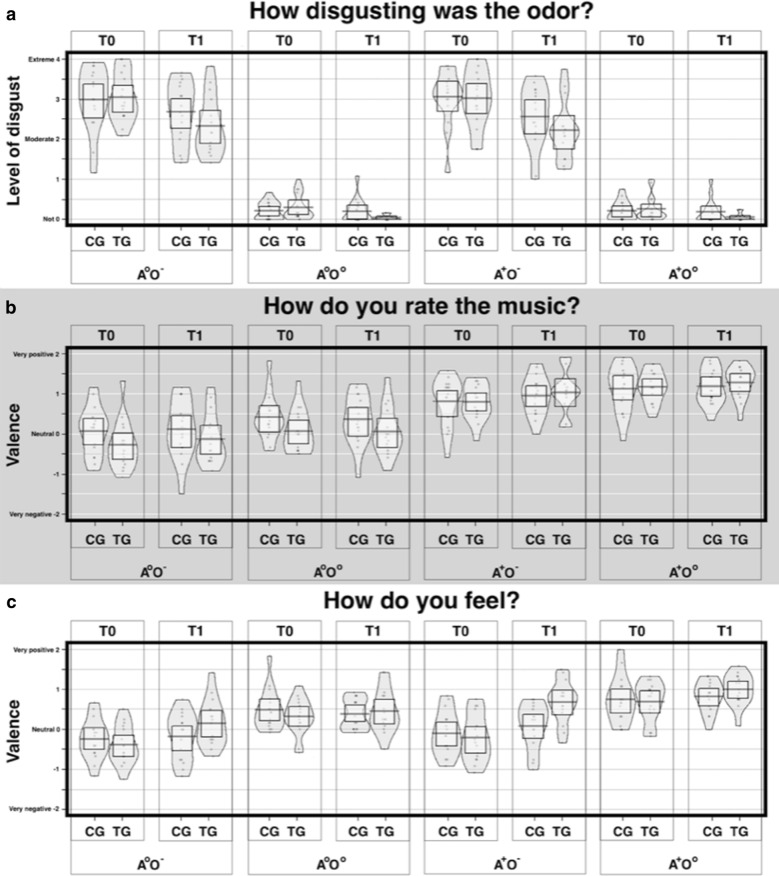
Schematic visualization of the experimental design. Exemplary trial (duration 32 s) out of 48 trials, presented in the fMRI task. Each trial began with the presentation of an auditory stimulus (duration 16 s, first second fade-in, last second fade-out). Four olfactory stimuli (duration 1 s each, interrupted by breaks of 2 s) were administered during the continuing presentation of the auditory stimulus. All four of the olfactory stimuli were either negative or neutral. The first olfactory pulse was administered jittered at random with a delay of 1.0, 1.5, 2.0, or 2.5 s relative to the beginning of the auditory stimulus, so always after the fade-in period was completed. The administration of the stimuli combination was followed by three ratings (duration 11 s, 3.66 s per rating) and a baseline period (duration 5 s), where a fixation cross was shown [13]

No other effects were significant, but two strong trends were observable. We detected a trend for an effect of time in general (F_1,30 =_ 3.96, p = 0.056; η_p_^2^ = 0.12). with a trend for an increase in valence ratings of the auditory stimuli. The second trend indicated an interaction between olfactory stimuli and time, O x T (F_1,30 =_ 3.93, p = 0.056; η_p_^2^ = 0.12) with a stronger increase in valence ratings in conditions with disgusting smells. We did not find any other significant effect on the valence ratings of the auditory stimuli (all p > 0.05). See Fig. 2b for an overview of the valence ratings of the auditory stimuli.

#### Ratings of emotional state

The two groups did not differ at baseline in emotional rating scores (all p > 0.05). Overall, a significant effect of the olfactory stimuli (F_1,30_ = 105.81, p < 0.001; η_p_^2^ = 0.78) and a significant effect of the auditory stimuli (F_1,31_ = 45.19, p < 0.001; η_p_^2^ = 0.60) were found on the ratings of the individual affective state. Under the same auditory condition, the emotional state was rated significantly more negative during negative compared to neutral olfactory stimulation (t = − 10.176, p < 0.001), while the effect of negative olfactory stimuli on emotional state was lower when combined with positive music A × O (F_1,30_ = 7.21, p = 0.012; η_p_^2^ = 0.19).

A significant general effect of Time (F_1,30_ = 17.67, p < 0.001; η_p_^2^ = 0.37), with a general increase in the general emotion well-being over all conditions was found. Furthermore, a significant interaction between the olfactory stimuli and Time, O x T (F_1,30_ = 11.91, p = 0.002; η_p_^2^ = 0.28) was found, which relates to a general stronger increase in well-being at the second measurement in the condition in which a disgusting smell was administered. The interaction between auditory stimuli and Time, A x T was also significant (F_1,30_ = 13.01, p = 0.001; η_p_^2^ = 0.30), with a stronger difference between the auditory stimuli at the second measurement. In relation to the training group, we observed a significant interaction of Time and Group, T x G (F_1,30_ = 10.92, p = 0.002; η_p_^2^ = 0.27), with only the group with music training showing an increase in emotion ratings and the no-training group remaining stable. Furthermore, we found a strong trend in the interaction between negative olfactory stimuli, Time, and Group, O x T x G (F_1,30_ = 3.38, p = 0.07; η_p_^2^ = 0.10), with the control group (CG) feeling emotionally worse during the occurrence of negative odours on the second measurement than the training group (TG). Despite the low sample size, we even found a trend that the emotional state during the occurrence of negative odours was differently modulated by the presence of positive auditory stimuli A x O x T x G (F_1,30_ = 3.69, p = 0.06; η_p_^2^ = 0.11). During the second measurement indeed, the musical training group felt significantly emotionally better during the occurrence of positive auditory and negative odours (A^+^ O^−^) than the control group (t = − 3.034, p = 0.005). Figure 1C shows an overview of the ratings of the emotional states.

### Imaging results

#### Main effect of positive auditory and negative olfactory stimulation

Main effects of the two stimuli were similar to the initial study of the first measurement [13], whereas no significant group differences after the musical training could be found in the imaging results.

#### Interaction of auditory and olfactory stimulation before and after the musical training

None of the main effects or differential contrast did reveal significant interaction with the training groups.

## Discussion

In our previous study we have shown that both auditory and olfactory stimuli are able to generate congruent emotions, but the pattern found in affectively rival stimulation suggested that implicit emotion regulation seems to be working uni-directionally in the case of negative olfaction paired with positive music: The music did not relieve the negative affect caused by the smell, but the smell tainting the enjoyment of the music and the overall well-being, which renders useful from an evolutionary perspective. We assumed that disgust of the olfactory stimuli and valence of the musical stimuli potentially reflect lower level (e.g. generation) emotional perception of the stimuli, while thinking about the current emotional state and giving to it a judgment reflects higher order regulatory processes. Relations with behavioural ratings that were found in the previous study, indicated that modulation of brain activation in these areas may also guide conscious labelling of emotional state and valence of external stimuli. Thus, as discussed earlier these areas may link integratory brain activation to emotional experience and therefore play a central role on implicit emotion regulation [13].

In the present training study, we aimed to investigate whether a three-week musical training is able to enhance implicit emotion regulation abilities on mood within an affective rivalry paradigm using stimuli of the auditory and olfactory system. We found that the musical training induced a behavioural change on the subjective affective state which is concordant with our initial hypothesis. In fact, only participants who received music training showed lower negative affective state when negative odours were paired with positive music. Such effect suggests that regular listening to music might increase implicit emotional regulatory abilities when unpleasant emotional conflictual contests occur. Passive listening to classical music twice per day might have led to a modulation of attention towards the musical stimuli presented during the experiment. The musical stimuli might have drawn attention more strongly, this might have had a ‘protective’ function from the impact of the negative olfactory stimulus. Only the training group did display significantly better subjective affective state as a result of getting negative olfactory stimulation while listening to positive music. The training might therefore in this group have led to a stronger implicit regulatory effect of positive auditory stimulation on the evaluation of the subjective affective state. However, the training did not induce any change at whole brain level. Additionally, we did not find any change in the activity of brain areas typically involved in emotional regulation processes, such as the dorsolateral prefrontal cortex (DLPFC; e. g. attentional processes), or in brain areas which indicate the necessity to regulate per se (ventrolateral prefrontal cortex; VLPFC) [34]. Despite these null results on whole brain level, the effect found in the behavioural measurements after the training indicates an enhanced implicit regulatory mechanism in a contest of daily-life implicit affective rivalry. These results are in line with those from systematic music therapy studies on clinical population [35–38]. However, to the authors’ knowledge, up to date no studies have investigated music-training effects on implicit emotion regulation processes in a contest of affective rivalry. If brain-function changes following musical training might lead to enhanced implicit emotional regulation skills, remains a question to be further investigated. One possible explanation of such brain-behavioural in-congruency is that the duration and/or intensity of our musical training was not intense enough to induce functional changes at brain level or that the training design was not sufficiently powered to detect subtle changes in brain activity in interaction with the training and stimulation. This seems in line with recent results from a music-based emotion regulation momentary assessment study in distressed participants, which indicate a significant music-related improvement in emotion regulation abilities only after two months of musical intervention (and not before) [39]. Another possible explanation might rely on the nature of implicit emotional regulatory changes per se. Emotion regulation processes might indeed change along the musical training, leading the participants to develop more conscious strategies (for instance from implicit to more explicit-controlled strategies) of emotional regulation that we, perhaps, were not able to catch with this specific fMRI paradigm (which is principally designed to investigate implicit emotional regulation brain processes during the paired presentation of the stimuli) [40]. Finally, we cannot exclude that behavioural changes might have been triggered by ‘social desirability’ effects on subjective affective ratings: participants in the training group—although not informed—may have assumed a purpose behind the training and if so, we cannot rule out the possibility that such an assumption may have influenced the affective ratings. In summary, the present study represents a first attempt to investigate the neuro-cognitive mechanisms of implicit emotion regulation during affective rivalry and offers a first base for future similar neuroimaging studies on [41] clinical populations.

### Limitations

We did not assess subjective ratings of unpaired auditory and olfactory stimuli; thus, we cannot be sure on valence of these stimuli. Nevertheless, we would argue, that the pairing with neutral stimulation can serve as a reasonable control condition and especially for positive music (paired with non-scented air) the values reflect unbiased reports. Furthermore, we cannot rule out the possibility that the differences rather reflect a valence effect, that is that negative stimulation wins over positive stimulation. We cannot refute this possible effect on the basis of our data, which would pose an interesting question for future research.

Participants may have practiced music as amateurs for many years more than one year before the study. This may have influenced the impact of the musical excerpts on their emotional state. As we are using very well-known musical excerpts, former amateurs may be more familiar with them and with musical stimuli in general, and this might diminish the effect of the music-listening training (as they were already trained beforehand), and thus reduce the experimental effect for those individuals. One year of musical inactivity will not wash out many years of training during childhood and adolescence. Musical training effects are known to persist [42, 43].

Although the auditory stimuli used in this study have shown to be capable of inducing affect-congruent emotions, subjectively selected musical pieces may lead to an enhanced effect, which could be a topic for future research. See also Blood et al. 2001 [44].

## Conclusions

In our study we demonstrate that a three-week musical training leads to a more positive affective state when negative odours are paired with positive music. However, a three-week musical training is not related to significant plastic changes. These results seem to suggest that implicit emotion regulation processes can be positively modulated by a regular music listening practice yet might only marginally influence related brain activation.

## Methods

### Participants and general procedure

Thirty-two volunteers, divided into a music intervention group (n = 16, mean age = 24.93 years, SEM = 0.80) and a non-intervention control group (n = 16, mean age = 25.25 years, SEM = 0.76), participated in the experiment. The mean educational level was 13 years of school (SEM = 0.1) for both the intervention group (SEM = 0.1) and the non-intervention group (SEM = 0.06). The participants and the task were identical to our previous study on affective rivalry [13], in which we only analysed the first measurement with collapsed groups. The participants did not listen to music more than 60 min per day and did not play or practice any musical instrument in the last 12 months, nor have they been musical professionals at any time. All participants were right-handed and had no history of neurological or psychiatric disorders or severe head trauma and no known abnormalities in olfactory or auditory function tested by the use of the semi-structured interview SKIDPIT [45].

Each participant was tested twice, one session at the beginning of the experiment and a second session 21 days after the first measurement. The experimental procedures and task employed in the two testing sessions were identical. During each of the two fMRI measurements we exposed the participants to unpleasant odours with an olfactometer, together with the auditory stimulation. Participants were instructed to actively listen to the pieces of music but were not asked to perform any kind of cognitive mood regulation. The music intervention group conducted a twenty-day musical training in-between the two measurements. Additionally, we did not inform the participants regardless of group about the aim of the study and asked them not to change their behavioural patterns during the experiment.

### Musical training

The music intervention or musical training consisted of passively listening to a selection of classical music, twice a day, in the morning and in the evening for about 15 min each session. The music was selected according to previous studies, that have shown its efficacy to successfully induce positive emotional states [19, 41, 46–52] (Table 2). The music pieces were merged into 20 sessions of approximately 15 min, whereby only a few shorter pieces occurred (at most) twice, in order to maintain a high level of variety. The sequence for the 20 morning sessions was randomised for each participant. For the evening sessions the reverse sequence was taken.

**Table 2:** Positive auditory material for the music training and fMRI task

Composer	Piece	Part	Classification
Bach, J.S	Brandenburg concerto no. 2 in F major	I. Allegro	BWV. 1047
Beethoven, L.v	Piano concert no. 4 in G major	I. Allegro moderato, II. Andante con moto, III. Rondo vivace	Op. 58
Beethoven, L.v	Symphony no. 6 in F major	I. Allegro ma non troppo, II. Andante molto mosso, III. Allegro, IV. Allegro, V. Allegretto	Op. 68
Beethoven, L.v	Symphony no. 3	I. Allegro con brio, II. Marcia funebre, III. Scherzo, IV. Finale	Op. 55
Bizet, G	Carmen Suite 2	Chanson du Toréador **( +)**	WD. 31
Mozart, W.A	Serenade no. 13 in G major	I. Allegro **( +)**, IV. Rondo Allegro **( +)**	K. 525
Mozart, W.A	Piano concert no. 23 in A major	III. Allegro	K. 488
Mozart, W.A	Divertimento in D major	I. Allegro, II. Andante, III. Presto **( +)**	K. 136
Mozart, W.A	Piano concert no. 27 in B flat	III. Rondo Allegro	K. 595
Mozart, W.A	Clarinet concert in A major	I. Allegro, II. Adagio, III. Rondo allegro	K. 622
Mozart, W.A	Ein musikalischer Spaß	I. Allegro, II. Menuetto, III. Adagio cantabile, IV. Presto	K. 522
Prokofjew, S	Peter and the Wolf	Peter in the Meadow **( +)**	Op. 67
Ravel, M	Le Tombeau de Couperin	IV. Rigaudon	
Saint-Saëns, C	Carnival of the Animals	Aviary, Finale **( +)**	R. 125
Schumann	Kinderszenen	Ritter vom Steckenpferd	Op. 15
Strauss Jr., J	Blue Danube Waltz		Op. 314
Strauss Sr., J	Radetzky March		Op. 228
Vivaldi, A	Concert in G major for two mandolins and orchestra	I. Allegro **( +)**, II. Andante, III. Allegro	RV. 532
Vivaldi, A	The four seasons: Concerto no. 3 in F major	I.A. Ballo e canto de villanelli, III.E. La caccia	RV. 293

The pieces were combined to 20 sequences with approximately 15 min length. Some shorter pieces were used more than once. The participants of the training group listened daily to the sequences in the mornings in randomised and in the evenings in backwards order. The pieces marked with (+) were used to generate the positive auditory stimuli presented in the fMRI task

The music was provided online through an internet server and additionally on a CD in case no internet was available. Each participant could log in with a separate username/password combination within a time frame in the morning and evening, the sessions were time-logged. The participants were explicitly asked not to do anything else while listening to the music, and at the end of each session they had to answer two pro forma questions about the music to maintain high levels of attention during the sessions.

### Auditory stimuli

The auditory stimuli were generated from musical scales and pieces previously shown to induce positive or neutral emotional states, then evaluated and selected in a separate pre-study on an independent set of subjects. Based on the ratings in the pre-study a set of 8 positive (Table 2) and 4 neutral auditory pieces (1. C major scale up, 2. C major scale down, 3. Adagio of Mozart’s Clarinet Concert and 4. Allegro Moderato of Beethoven’s Piano Concert no. 4) were selected for the presentation during the fMRI task. The volume of the sequences was levelled and adjusted to each participant individually before the experiment, to assure that the sound was clearly perceivable. Details about the pre-study and generating of the auditory stimuli are presented in our previous study [13]. The music bits taken for the positive auditory stimuli were all also parts of the music pieces selected for the training.

### Olfactory stimuli

The participants were exposed to one olfactory condition (the unpleasant odour H_2_S –hydrosulphide- in nitrogen) and a neutral baseline condition, during which the regular ambient airflow was held constant with no odour superimposed [13]. The application was unirhinally on the right side, which shows better results regarding the BOLD responses as described in several studies [53–55]. For the stimulus presentation a Burghart OM4 olfactometer [56] was used.

### fMRI task and valence ratings

One session included 48 blocks, during each block, in the first 16 s the auditory in combination with the olfactory stimuli were presented, followed by three valence ratings with a total duration of 11 s and finally a baseline period of 5 s (Fig. 2). During the valence ratings, the participant had to indicate on a 5-point scale a) how disgusting they would rate the smell (0 = not at all to 5 = extremely), b) how they would rate the music (0 = very negative to 5 = very positive; 2.5 = neutral), and c) how they currently felt (0 = very bad to 5 = very good; 2.5 = neutral). During stimulus presentation and baseline period a fixation cross was visible. We assumed that disgust of the olfactory stimuli and valence of the musical stimuli potentially reflect conceptually lower level (e.g. generation) emotional perception of the stimuli, while thinking about the current emotional state and giving to it a judgment reflects conceptually higher order regulatory processes.

The auditory stimuli (A) were either neutral (A^0^) or positive (A^+^), were faded in during the first and out during the last second by linear volume progression/regression.

Simultaneously either normal air as neutral (O^0^) or H_2_S as negative (O^−^) olfactory condition (O) was presented. To minimise habituation effects H_2_S was applied in 4 pulses with duration of 1 s each, interrupted by breaks of 2 s in between, a procedure which has been successfully applied in previous studies [17, 57, 58]. The beginning of the first olfactory pulse was jittered at random relative to the beginning of each auditory stimulus. For the visual presentation in the scanner including the ratings the software Presentation (Neurobehavioural Systems Inc., Berkeley, CA, USA) was used. The participants gave their responses by moving a cursor to the desired location via button boxes (LumiTouch, Photon Control, Burnaby, Canada). Each combination of the two different stimuli (A^0^O^−^, A^0^O^0^, A^+^O^−^, A^+^O^0^) was presented 12 times in randomised order of appearance. The same procedures were applied during the second fMRI measurement 21 days later.

### fMRI data acquisition

Functional imaging was performed on a 3 T Trio MR Scanner (Siemens Medical Systems, Erlangen, Germany) using echo-planar imaging (EPI) sensitive to BOLD contrast (whole brain, T2*, voxel size: 3.4 × 3.4 × 3.3 mm^3^, matrix size 64 × 64, field of view [FoV] = 220 mm^2^, 36 axial slices, slice gap = 0.3 mm, acquisition orientation: ascending, echo time [TE] = 30 ms, repetition time [TR] = 2 s, flip angle [α] = 77°, approx. 780 volumes, slice orientation: AC-PC).

### Statistical data analysis

#### Behavioural data

All analyses were performed with SPSS Software (IBM SPSS for Statistic, version 24). Repeated measures analyses of variance (rmANOVA) were calculated for the three dependent variables disgust, music valence, and emotional state ratings, using two factors: Auditory stimulation (with two levels: A^0^, A^+^) and olfactory stimulation (with two levels: O^0^, O^−^). This model generated a 2 × 2 design per session. To investigate the effect of the musical training on the subjective affective state two factors were added: a factor time (T) was used as a within subjects factor with two levels (T0: before training, T1: after training), and a factor group (G) was used as between subjects factor with two levels (control group, CG: no musical training; training group, TG: with musical training). Thus, a 2 × 2x2 × 2 repeated measures ANOVA was implemented in SPSS. Significant interaction effects were decomposed by post-hoc paired-sample t-tests when applicable. Greenhouse–Geisser corrected p-values were used. Note that SPSS implements a mixed-effects model that account for subject as random effect.

#### fMRI data processing

The functional and anatomical images were preprocessed and analysed using the FMRIB Software Library (FSL; Oxford Centre for Functional Magnetic Resonance Imaging of the Brain, University of Oxford, UK; www.fmrib.ox.ac.uk/fsl) [59]. The first three volumes of each functional time series were discarded to disregard magnetization effects and initial transient signal changes. Functional images were spatially smoothed with a 5 mm full width at half-maximum Gaussian kernel to reduce inter-subject variability. Further preprocessing steps include three-dimensional Movement Correction using FMRIB’s Linear Image Registration Tool (MCFLIRT) [60]. Furthermore, Independent Component Analysis based Automatic Removal of Motion Artifacts (ICA-AROMA) was used for denoising [61, 62]. By linear regression of ICA components, AROMA identified components as noise and (non-aggressively) regresses out the time courses of these components. Subsequently, a high pass filter of 100 s was administered. Prior to group analyses, individual functional images were normalised to MNI space in a two-step procedure combining linear and non-linear registration.

For the first level analysis four different exploratory variables (EVs) (A^0^O^−^, A^0^O^0^, A^+^O^−^, A^+^O^0^) were defined (block design) in the general linear model. Each block was modelled as lasting 16 s (onset to end) and labelled according to the underlying combination of olfactory and auditory stimuli. The onset functions and durations were convolved with the canonical hemodynamic response function (HRF). Additional nuisance regressors for instructions, any displays of no interest, as well as eventual confounding order-effects in the presentation in olfactory/auditory stimuli between the first (T0) and second fMRI session (T1) were added as covariates of non-interest. We built contrasts from these parameter estimates. The contrasts reflected the main effect per condition (A^0^O^−^, A^0^O^0^, A^+^O^−^, A^+^O^0^), the main effect of positive auditory and negative olfactory stimulation respectively (A + and O−) as well differential effects (positive auditory minus neutral auditory and negative olfactory minus neutral olfactory). In a fixed effects analysis per subject, the repeated measurements for all these contrasts were modelled on an intermediate level (average effect collapsed over conditions and difference between the measurement time-points). The parameters estimated from this intermediate level were entered into a two-sample t-test on the group level.

Thereby, we examined group differences’ main effects of conditions, differential contrasts as well as interactions. Of these contrasts, the interaction of time and group difference in brain activity to condition A^+^O^−^ was the one most directly tested the hypothesis of interaction of training with automatic emotion regulation. Furthermore, group differences on interaction terms would indicate modulation of integration and appraisal processes.

## Data Availability

The datasets generated during and/or analysed during the current study are not publicly available due to lack of consent by participants. When the ethics approval for the current study was submitted, data sharing was not standard. The ethics committee of the Medical School, RWTH Aachen University, Germany subsequently decided that if participants were not asked to grant approval for data sharing, data sharing would not be allowed.

## Abbreviations

ANOVA: Analysis of variance
AROMA: Automatic Removal of Motion Artifacts
BOLD: Blood oxygen level dependant
CD: Compact disc
DLPFC: Dorsolateral prefrontal cortex
e.g.: Exempli gratia
EPI: Echo-planar imaging
EV: Exploratory variables
fMRI: Functional magnet resonance imaging
FMRIB: Functional Magnetic Resonance Imaging of the Brain
FSL: FMRIB software library
HRF: Hemodynamic response function
ICA: Independent Component Analysis
MCFLIRT: Movement Correction using FMRIB’s Linear Image Registration Tool
MNI: Montreal Neurological Institute
rmANOVA: Repeated measures analysis of variance
SPSS: Statistical Products and Service Solutions
VLPFC: Ventrolateral prefrontal cortex
